# Effect of Diamine Monomers with Varied Backbone Structures on Dielectric and Other Comprehensive Properties of Fluorinated Polyimide Films

**DOI:** 10.3390/polym17111505

**Published:** 2025-05-28

**Authors:** Wenhao Xu, Xiaojie He, Yu Zhou, Lan Jiang, Weiyou Yang, Qinghua Lu, Peng Xiao

**Affiliations:** 1 Institute of Micro/Nano Materials and Devices, Ningbo University of Technology, Ningbo 315211, China; 2 School of Chemical Science and Engineering, Tongji University, Siping Road No. 1239, Shanghai 200092, China; 3 State Key Laboratory of Micro-Nano Engineering Science, Shanghai Jiao Tong University, Dongchuan Road No. 800, Shanghai 200240, China

**Keywords:** fluorinated polyimide, dielectric properties, optical transparency, molecular simulation, structure–property relationship

## Abstract

Fluorinated polyimide (FPI), renowned for its exceptional low-dielectric properties, colorless transparency, high-temperature resistance, and flexibility, has emerged as an ideal material for addressing challenges in 5G/6G high-frequency signal transmission and flexible electronic substrates. Nevertheless, the structure–property relationship between molecular architectures and the dielectric characteristics of FPI films remains insufficiently understood, necessitating urgent elucidation of the underlying mechanisms. In this study, a diamine monomer containing bis-amide bonds, 4-amino-N-{4-[(4-aminobenzoyl)amino]phenyl}benzamide (PABA), was synthesized. Subsequently, six FPI films (FPAIs, FPEIs, and FPEsIs) with distinct structural features were prepared through homopolymerization of PABA and five other diamines (containing amide bonds, ether, and ester groups) with fluorinated dianhydride (6FDA). Systematic characterization of thermal, mechanical, optical, and dielectric properties revealed that these films exhibit excellent thermal stability (*T*_g_: 296–388 °C), mechanical strength (*σ*: 152.5–248.1 MPa, *E*: 2.1–3.4 GPa), and optical transparency (*T*_550 nm_: 82–86%). Notably, they demonstrated a low dielectric constant (*D*_k_ as low as 2.8) and dielectric loss (*D*_f_ down to 0.002) under both low- and high-frequency electric fields. Furthermore, molecular dynamics simulations and quantum chemical were employed to calculate critical physical parameters and HOMO–LUMO energy levels of the six FPIs. This computational analysis provides deeper insights into the structure–performance correlations governing dielectric behavior and optical transparency in FPIs. The findings establish valuable theoretical guidance for designing advanced PI films with tailored dielectric properties and high transparency.

## 1. Introduction

With the rapid advancement of artificial intelligence, the Internet of Things (IoT) [1], and 5G/6G communication technologies [2,3,4], the demand for interlayer insulation materials in electronic devices continues to grow. Simultaneously, as large-scale integrated circuits evolve toward miniaturization, high integration, and flexibility, there is an increasing need to enhance signal transmission speed [5,6,7]. Due to its outstanding comprehensive properties, including excellent thermal stability, chemical stability, and mechanical performance, as well as its favorable structural design and formability, PI has found widespread application in fields such as electrical insulation, microelectronics, flexible substrates, and aerospace [8,9,10]. Conventional PI films typically exhibit a dielectric constant (*D*_k_) of approximately 3.5 and a dielectric loss (*D*_f_) of around 0.01 [11,12,13]. However, the demands of high integration and high-speed signal transmission often lead to increased operating temperatures and greater signal losses, thereby affecting device performance and longevity [14,15]. To ensure fast and stable signal transmission, insulation materials must not only possess good mechanical and thermal properties but also exhibit low *D*_k_ and *D*_f_ values [16,17,18]. Consequently, the design and synthesis of next-generation low-dielectric PI materials have become a key research focus in microelectronics and materials science.

Fluorine (F) modification is an effective approach to lowering the *D*_k_ of PI [19,20,21]. The strong electron-withdrawing ability of F (with the highest electronegativity of 4.0) allows it to capture electrons and exhibit weak polarization tendencies. Incorporating F into the PI molecular structure effectively reduces molecular polarization [22]. Additionally, F disrupts the structural regularity of polymer chains and increases the free volume, further reducing the number of polarizable molecular units per unit volume [23]. Moreover, F incorporation significantly lowers the water absorption of the polymer, thereby enhancing dielectric stability. Typically, F is introduced into the PI structure via two primary substituents: F and trifluoromethyl (CF_3_), leading to the synthesis of FPI. Ando et al. reported a strategy involving the substitution of benzene ring hydrogen atoms with F, leading to a series of FPIs with *D*_k_ values as low as 2.73 at 10 GHz (23 ± 1 °C, 30% RH) [24]. Scola et al. synthesized FPI films by introducing perfluorinated benzene side groups into dianhydrides, achieving a *D*_k_ as low as 2.01 at 1 kHz (40–60% RH) [25]. Comparatively, most FPIs incorporate F into the PI backbone via the “CF_3_” functional group. Huang et al. developed a novel perfluorocyclobutyl-phenyl-ether-based PI, which exhibited exceptional moisture resistance and maintained ultra-low-dielectric stability (*D*_k_ < 2.5) even under prolonged high-humidity conditions [26]. Li et al. synthesized three FPIs via the homopolymerization of 6FDA with three different CF_3_-substituted diamines, all of which demonstrated ultra-low *D*_k_ values (~1.84 at 10^4^ Hz) [27]. Zhang et al. introduced a rigid non-planar fluorenyl diamine with a CF_3_-phenyl group into the PI main chain, reducing molecular chain polarization and increasing free volume, yielding an FPI film with an ultra-low *D*_k_ (~1.93) [28]. Chen et al. and our research group investigated the correlation between F content (F%) and *D*_k_ in PI, revealing a strong correlation coefficient of 0.98 [29]. Overall, fluorination is an effective strategy for reducing *D*_k_; however, it is noteworthy that an optimal F% range of 25–37% is critical, as excessive F incorporation beyond this range has minimal impact on further *D*_k_ reduction.

The *D*_f_ in polymers primarily originates from polarization loss and conduction loss. Polarization loss occurs during the polarization and depolarization processes of polymer dielectrics. At high external electric field frequencies, the internal polarization process of the dielectric cannot synchronize with the field variations, resulting in hysteresis effects and polarization loss. Conduction loss, on the other hand, is induced by leakage currents and can be classified into bulk-limited conduction and electrode-limited conduction. In the low-frequency range, dipoles within the material have sufficient response time, making conduction loss dominant and largely independent of frequency. However, under high-frequency electric fields, the dipole response time shortens, leading to a significant increase in polarization loss. At this stage, the *D*_f_ is jointly determined by conduction and polarization losses and increases with frequency. Liu et al. incorporated a rigid aromatic ester dianhydride monomer (TAHQ, terephthalate-based dianhydride) into the PI backbone and optimized the imidization process to induce liquid crystalline structures, ultimately obtaining liquid crystalline PI films with extremely low *D*_f_ values (0.00365 at 1 MHz, 0.00184 at 10 GHz) [30]. Our research group has previously reported the introduction of ether and ester groups into PI structures to significantly reduce the *D*_f_ by modulating polymer orientation, crystallinity, and the rigidity–flexibility balance [31,32,33]. While FPI exhibits substantial advantages in reducing *D*_k_, its *D*_f_ behavior differs, showing a weaker correlation with F% but a stronger dependence on other structural factors. To meet the requirements of high-speed data transmission in 5G/6G high-frequency communications, a more comprehensive understanding of the structure–property relationships governing *D*_f_ and *D*_k_ in FPI films is urgently needed.

In this study, we synthesized a diamine monomer, 4-amino-N-{4-[(4-aminobenzoyl)amino]phenyl}benzamide (PABA), and subsequently homopolymerized it with six different diamines containing amide, ester, or ether linkages using 4,4′-(hexafluoroisopropylidene)phthalic anhydride (6FDA) as the dianhydride monomer. This process resulted in the fabrication of six FPIs (FPAIs, FPEIs, and FPEsIs) with distinct structural characteristics. A comprehensive characterization of these films demonstrated excellent thermal, mechanical, and optical properties. Furthermore, we systematically investigated their dielectric properties, including *D*_k_ and *D*_f_, under low- and high-frequency conditions and various environmental settings. Finally, molecular dynamics (MD) simulations and quantum chemical calculation were employed to elucidate the structure–property relationships between different functional groups and the dielectric and optical performance of these FPIs. The findings of this study provide valuable insights for the rational design and synthesis of low-*D*_k_, low-*D*_f_, and highly transparent PI materials.

## 2. Materials and Methods

### 2.1. Materials

4,4′-(Hexafluoroisopropylidene)diphthalic anhydride (6FDA), 4,4′-diaminobenzanilide (DABA), 4,4′-oxydianiline (ODA), *p*-phenylenediamine (PDA) 1,4-bis(4-aminophenoxy)benzene (TPE), *p*-aminophenyl *p*-aminobenzoate (APAB), [4-(4-aminobenzoyl)oxyphenyl] 4-aminobenzoate (ABHQ), and bis(4-aminophenyl) terephthalate (BATP) were purchased from Tianjin Zhongtai Chemical Technology Co., Ltd. (Tianjin, China) with purity > 99.5%. *N*, *N*-dimethylformamide (DMF), and *N*, *N*-Dimethylacetamide (DMAc) was obtained from J&K Scientific Ltd. (Beijing, China) (purity 99.8%, water content < 50 ppm). Deuterated dimethyl sulfoxide (DMSO-*d_6_*), tetrahydrofuran (THF), pyridine (99.5%), palladium on carbon (Pd/C, 10%), 4-nitrobenzoyl chloride (99.8%), anhydrous ethanol (EtOH), and petroleum ether (PE) were sourced from Adamas Reagent Co., Ltd. (Shanghai, China). All reagents were used as received without further purification.

### 2.2. Characterization

The characterization of nuclear magnetic resonance hydrogen spectra (^1^H NMR) was conducted using a Bruker AVANCE III HD 600 MHz spectrometer (Ettlingen, Germany). A sample of 20–40 mg was weighed into a tube, dissolved in 0.5 mL of deuterated solvent (DMSO-*d*_6_), and subsequently analyzed. The Fourier transform infrared (FT-IR) spectra of the PI films were obtained using a Thermo Fisher IS-5 spectrometer (Waltham, WA, USA) in transmission mode. The samples were directly placed on a barium fluoride (BaF_2_) substrate and measured under ambient dry conditions within a spectral range of 4000–500 cm^−1^, with 16 scans per measurement. The molecular weight of the poly(amic acid) (PAA) precursor of PI was determined using a TOSOH HLC-8320 gel permeation chromatography (GPC) system (Tokyo, Japan). Polystyrene (PS) was used as the calibration standard, and chromatography-grade DMAc containing lithium bromide (0.03 M) and phosphoric acid (0.03 M) was used as the eluent. The glass transition temperature (*T*_g_) of the films was measured using a TA Instruments Q800 dynamic mechanical analyzer (DMA) (New Castle, DE, USA). The films were cut into rectangular strips (3.5 mm in width) using a cutter, and the test was conducted in dynamic tensile mode at a heating rate of 5 °C/min over a temperature range of 30–400 °C, with a frequency of 1 Hz under a nitrogen atmosphere. The coefficient of thermal expansion (CTE) of the films was determined using a TA Instruments Q400 thermomechanical analyzer (TMA) (USA). In tensile mode, the sample was first heated to 250 °C (below its *T*_g_) to eliminate thermal history. The measurement was then performed under a nitrogen atmosphere at a heating rate of 5 °C/min over a temperature range of 25–400 °C, with a fixed load of 0.005 N. The thermal stability of the films was analyzed using a PerkinElmer Pyris Discovery 550 thermogravimetric analyzer (TGA) (Shelton, CT, USA). Approximately 10 mg of each sample was tested under a nitrogen atmosphere. Initially, the sample was heated at a rate of 20 °C/min to 110 °C and held for 20 min to remove moisture. The temperature was then lowered to 50 °C before a second heating cycle was performed at a rate of 10 °C/min within a temperature range of 50–800 °C. The mechanical properties of the films were evaluated using a Zhuhai Sansi Taijie electronic universal testing machine (Zhuhai, China). The tensile properties were measured according to the ASTM standard, with a tensile rate of 5 mm/min. The films were cut into rectangular strips (10 mm in width) before testing. At least six specimens were tested for each sample, and the average value was reported as the final result. The high-frequency dielectric properties of the films were measured at 25 °C and 40% relative humidity using a Keysight PNA N5227B vector network analyzer (Santa Rosa, CA, USA). The split cylindrical resonator method was employed, utilizing a resonant cavity fixture (Keysight 85072A) with the TE_011_ resonant mode to determine the *D*_k_ and *D*_f_ at frequencies of 10, 40, and 60 GHz. The low-frequency dielectric properties of the films were assessed at 25 °C and 40% relative humidity (RH). The film thickness was first measured using a micrometer with a precision of 0.001 mm, followed by silver sputtering on both sides as a pre-treatment. Subsequently, the *D*_k_ and *D*_f_ were measured over a frequency range of 10^2^–10^6^ Hz using an Agilent E4980A precision LCR meter (Santa Clara, CA, USA). The optical transmittance and cutoff wavelength of the films were determined using a Shimadzu UV-2600 spectrophotometer (Kyoto, Japan) in transmission mode. The films were directly affixed to the detection window for measurement over a wavelength range of 200–800 nm, with a film thickness of 30–50 μm. The colorimetric values of the PI films were measured using a PerkinElmer Lambda 950 UV/Vis/NIR spectrophotometer (USA). The CIELAB and Yellowness Index (YI) values were obtained under a standard CIE D65 light source with a 10° observer angle. During moisture uptake evaluations, FPI films preconditioned at 80 °C for two hours were submerged in 25 °C deionized water for 48 h. Following immersion, surface moisture was removed from the retrieved specimens using absorbent paper, after which their weights were promptly recorded. The percentage of water absorption (*A*) was determined through the equation: $A=\frac{m_{2}-m_{1}}{m_{1}}\times 100\%$, where *m*_1_ and *m*_2_ represent the initial and post-hydration mass of the films, respectively.

### 2.3. Monomer Synthesis

**Scheme 1:**
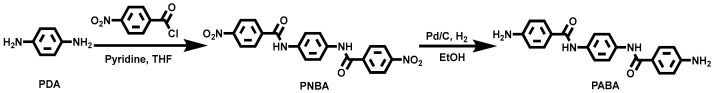
Synthetic route of PABA.

Synthesis of *N*, *N*′-(1,4-phenylene)bis(4-nitrobenzamide) (PNBA). Under a nitrogen atmosphere, 4-nitrobenzoyl chloride was dissolved in anhydrous THF (48 mL) in a 250 mL three-necked round-bottom flask. In a separate single-necked flask, *p*-PDA (3.3 g, 30.4 mmol) and pyridine (8.4 mL) were dissolved in THF (54 mL). Using a constant-pressure dropping funnel, the PDA and pyridine solution was slowly added dropwise to the 4-nitrobenzoyl chloride solution at 0 °C (Scheme 1). The reaction mixture was then stirred at room temperature for 24 h, and the reaction progress was monitored using thin-layer chromatography (TLC). Upon completion, 150 mL of deionized water was added to the reaction mixture, and the resulting solid was collected by filtration, yielding the crude dinitro compound. The crude product was washed three times with THF and subsequently dried under vacuum at 100 °C for 12 h to obtain the pure dinitro compound (14.7 g, 89% yield). ^1^H NMR (600 MHz, DMSO-*d*_6_): *δ* 10.61 (s, 2H, N-H), 8.38 (d, *J* = 1.2 Hz, 4H, Ar-H), 8.20 (d, *J* = 0.6 Hz, 4H, Ar-H), 7.80 (d, *J* = 0.6 Hz, 4H, Ar-H) ppm.

Synthesis of 4-Amino-N-{4-[(4-aminobenzoyl)amino]phenyl}benzamide (PABA). The dinitro compound (10.0 g, 18.4 mmol) was dissolved in DMF (100 mL), and 0.5 g of Pd/C (10 wt.%) was added as a catalyst (Scheme 1). The mixture was stirred under a hydrogen atmosphere for 12 h, with the reaction progress monitored by TLC. Upon completion, the Pd/C catalyst was removed by filtration from the solution. The filtrate was poured into an excess amount of water, resulting in the precipitation of a white solid. The precipitate was collected by filtration, recrystallized from a mixed solvent of PE and THF (1/10), and dried under vacuum to obtain PABA (7.5 g, 84% yield). ^1^H NMR (600 MHz, DMSO-*d*_6_) *δ* 9.73 (s, 2H, N-H), 7.72 (d, *J* = 1.2Hz, 4H, Ar-H), 7.67 (s, 4H, Ar-H), 6.60 (d, *J* = 0.6Hz, 4H, Ar-H), 5.75 (s, 4H, NH_2_) ppm.

### 2.4. Preparation of Polyimide Films

All six types of PI films were prepared using a two-step thermal imidization method. A 100 mL three-necked flask and a polytetrafluoroethylene (PTFE) stirring paddle were placed in a forced-air oven at 80 °C for approximately 10 min. After removal, they were transferred to a mechanical stirring apparatus, connected to a nitrogen gas inlet, and purged with nitrogen. At room temperature, DABA (0.9084 g, 4.0 mmol) was added to the flask, followed by the addition of 10 mL of DMAc. The mixture was stirred until the diamine was completely dissolved. Subsequently, dianhydride 6FDA (2.1867 g, 4.0 mmol) was added, along with an additional 2 mL of DMAc, and the reaction was stirred continuously for 12 h. The resulting product was a pale yellow, transparent, and viscous PAA solution. The PAA solution was then placed in a vacuum oven at room temperature for degassing for more than 10 h in preparation for the next step.

The PI films were fabricated using a solution-casting method. Under a controlled cleanroom environment with humidity maintained below 35%, a clean and dry glass plate was placed onto an automatic film coater. The degassed PAA solution was evenly poured onto the glass plate, and the coating knife was adjusted to a height of 500 μm. The film was cast at a coating speed of 5 mm/min, ensuring uniform deposition of the polymer solution onto the glass substrate. The coated glass plate was then transferred to a vacuum oven, where it was subjected to stepwise solvent removal at 80 °C, 100 °C, and 120 °C for 1 h at each temperature. Subsequently, the film was transferred to a high-temperature oven under a nitrogen atmosphere and thermally imidized at 150 °C, 200 °C, 250 °C, and 300 °C, each for 1 h, with a heating rate of 5 °C/min. After the thermal treatment, the oven was allowed to cool to below 50 °C. The glass plate containing the FPI film was then immersed in hot water to facilitate film detachment. The detached film was rinsed thoroughly with ultra-pure water and dried in a forced-air oven at 80 °C for 1 h. The final FPI films, with a thickness of approximately 40–50 μm and nearly transparent appearance, were sealed in self-locking plastic bags for further characterization.

## 3. Results and Discussion

### 3.1. Monomer Syntheses

**Figure 1:**
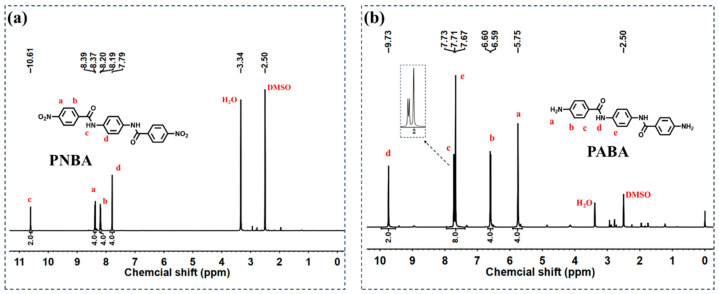
(**a**) 1H NMR spectra of PNBA; (**b**) ^1^H NMR spectra of PABA.

The chemical structures of PNBA and PABA were confirmed by ^1^H NMR spectroscopy. Figure 1a,b present the ^1^H NMR spectra of PNBA and PABA, respectively. In Figure 1a, the singlet signal at 10.61 ppm corresponds to the proton signal of the amide bond in the PNBA structure (labeled as c). The three pairs of doublet signals appearing in the range of 8.39–7.79 ppm are attributed to the aromatic protons of PNBA, corresponding to positions a, b, and d. Similarly, in Figure 1b, the singlet peak at 9.73 ppm is assigned to the proton signal of the amide bond in the diamine structure of PABA (labeled as d). The three sets of signals in the range of 7.73–6.59 ppm correspond to the aromatic protons at positions c, e, and b in PABA, while the peak at 5.75 ppm is attributed to the protons of the two amino groups. These results confirm the successful synthesis of the intermediate PNBA and the diamine PABA, both with high yield and purity.

### 3.2. Characterization of Polyimides

**Scheme 2:**
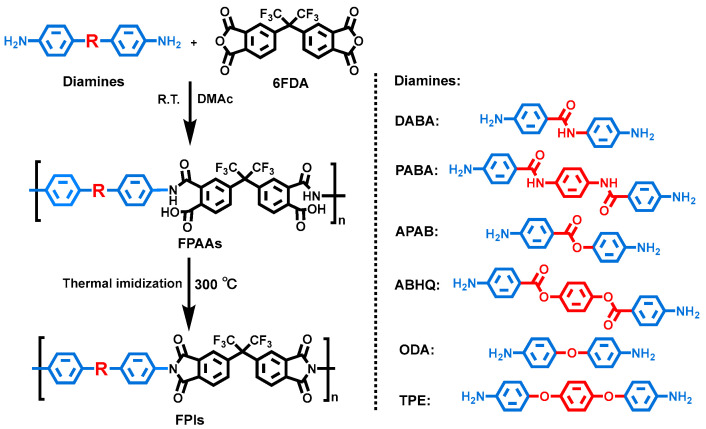
General synthesis procedure for FPIs.

A series of FPI films were prepared via thermal imidization by homopolymerizing six diamine with 6FDA, as illustrated in Scheme 2. The molecular weights of the precursor PAA were determined by GPC, and the results are summarized in Table 1. The weight-averaged molecular weight (*M*_w_) ranged from 9.2 × 10^4^ to 15.2 × 10^4^ g/mol, with a polydispersity index (PDI) of 1.9–2.4, indicating that all polymers possessed sufficiently high molecular weights and good molecular weight distributions, enabling the formation of tough FPI films.

**Figure 2:**
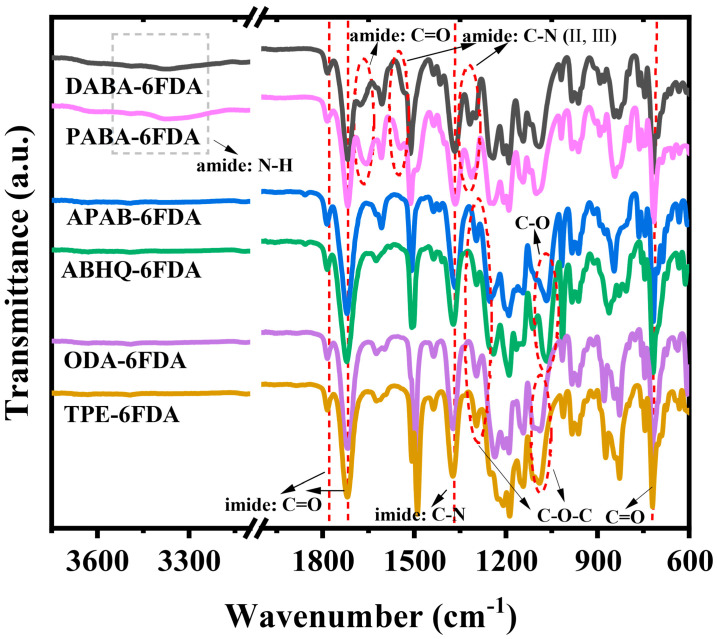
FT-IR spectra of the FPI films.

The chemical structures of the PI films were characterized by FT-IR spectroscopy, and the corresponding infrared spectra are shown in Figure 2. The FT-IR spectra of DABA-6FDA and PABA-6FDA exhibit a broad absorption band in the range of 3241–3552 cm^−1^, attributed to the N-H stretching vibration of the amide bonds. The characteristic peaks at 1784 cm^−1^, 1718 cm^−1^, and 721 cm^−1^ correspond to the asymmetric stretching, symmetric stretching, and bending vibrations of the C=O groups in the imide rings, respectively. Additionally, the peaks around 1658 cm^−1^ in the spectra of DABA-6FDA and PABA-6FDA are assigned to the C=O stretching vibration of amide bonds. The absorption band near 1374 cm^−1^ corresponds to the C-N stretching vibration of the imide rings. Furthermore, the peaks at 1545 cm^−1^ and 1318 cm^−1^ are attributed to the coupled in-plane bending (II band) and out-of-plane bending (III band) vibrations of the C-N and N-H bonds in the amide groups. For APAB-6FDA and ABHQ-6FDA, the peaks at 1296 cm^−1^ and 1065 cm^−1^ correspond to the asymmetric stretching vibration of the C-O-C ether groups and the single-bond C-O stretching vibration, respectively. Similarly, for ODA-6FDA and TPE-6FDA, the absorption peaks at 1295 cm^−1^ and 1089 cm^−1^ are assigned to the asymmetric and symmetric stretching vibrations of the C-O-C ether groups, respectively. Apart from PABA-6FDA and DABA-6FDA, which inherently contain amide bonds, no residual amide bond peaks were observed around 1650 cm^−1^ in the spectra of the other polymers, indicating the complete imidization of the polymers. These results confirm the successful synthesis of the target FPI films with fully imidized structures.

### 3.3. Solubility and Water Absorption of FPIs

The solubility properties of polymers are critical for their processability and potential applications. The solubility data for the six synthesized FPIs with different functional groups are summarized in Table S1. The FPEsIs (ABHQ-6FDA and APAB-6FDA) exhibited poor solubility, which can be attributed to the fact that ester groups act as mesogenic units [34].The presence of ester groups increases the crystallinity of the polymer, leading to more ordered molecular chain packing, which prevents solvent molecules from penetrating between the polymer chains. In contrast, the FPAIs (DABA-6FDA and PABA-6FDA) and FPEIs (ODA-6FDA and TPE-6FDA) exhibited good solubility in both highly polar and less polar solvents. This improved solubility can be attributed to the presence of the hexafluoroisopropylidene (-C(CF_3_)_2_-) unit, a bulky and twisted structural moiety, which effectively disrupts the close molecular packing of the FPI chains. This disruption results in lower crystallinity and enhanced solvent affinity, thereby significantly improving the solubility of these FPIs.

**Figure 3:**
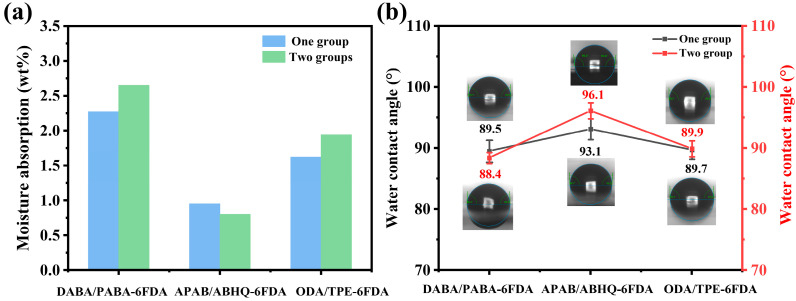
(**a**) Comparison of FPI films’ water absorption; (**b**) water CA for FPI films.

PIs typically exhibit high water absorption, primarily due to the presence of highly polar imide rings in their backbone. Therefore, understanding water absorption behavior is crucial for evaluating its impact on the dielectric properties of PIs. The water absorption of the six FPI films was measured, and the results are summarized in Table 1 and Figure 3a. The incorporation of ester groups, which are low-polarity and hydrophobic, significantly reduces the water absorption of FPI films. As a result, the FPEsIs (APAB-6FDA and ABHQ-6FDA) exhibited much lower water absorption compared to the other four FPIs without ester groups, with values consistently below 1%. Additionally, APAB-6FDA showed higher water absorption than ABHQ-6FDA, suggesting that a higher ester content leads to lower water uptake. Conversely, the FPAIs (DABA-6FDA and PABA-6FDA) and FPEIs (ODA-6FDA and TPE-6FDA) exhibited higher water absorption due to their hydrophilic nature, which allows them to form hydrogen bonds with water molecules. Moreover, in terms of hydrophilicity, amide bonds are more hydrophilic than ether linkages. Consequently, the FPAIs exhibited higher water absorption than the FPEIs.

The wettability of polymer films is another critical factor influencing their hydrophilicity and is closely related to dielectric performance. The wettability of the six FPI films was evaluated by measuring their static water contact angles (CA), as shown in Figure 3b and listed in Table 1. The results indicate that the PEsIs exhibited a CA greater than 90°, whereas the FPEIs and FPAIs showed a CA below 90°. Specifically, the water CA of the APAB-6FDA and ABHQ-6FDA films were 93.1° and 96.1°, respectively, demonstrating that an increase in ester content leads to a larger CA. On the other hand, the water CA of the PABA-6FDA and DABA-6FDA films were 88.4° and 89.5°, while those of ODA-6FDA and TPE-6FDA were 89.7° and 89.9°, indicating that the FPAIs exhibited higher wettability than the FPEIs. In summary, incorporating ester groups into the PI backbone effectively reduces the film’s wettability, which in turn helps lower the *D*_k_ of FPI films.

### 3.4. Dielectric Properties

The *D*_k_ and *D*_f_ of six FPI films with different functional groups were measured at 10, 40, and 60 GHz under conditions of 25 °C and 40% RH, with the results summarized in Table 2. The FPAIs (DABA-6FDA and PABA-6FDA) exhibited relatively high *D*_k_ values, approximately 3.2 and 3.7, respectively, with *D*_f_ values of 0.009 and 0.012. A comparison between DABA-6FDA and PABA-6FDA showed that as the amide content increased, both *D*_k_ and *D*_f_ increased significantly. According to the Clausius–Mossotti equation, $\frac{\varepsilon -1}{\varepsilon +2}=\frac{N\alpha }{3\varepsilon_{0}}$, *ε* is the *D*_k_, *N* is the dipole density, *α* is the polarizability, and *ε*_0_ is the permittivity of free space (a constant) [35]. An increase in the amide content directly enhances both *N* and *α*, leading to a higher *D*_k_. To further investigate the mechanism underlying the influence of different functional groups on the dielectric properties of PIs, molecular simulations were performed on the six FPI molecular chains, and the relevant physical parameters were obtained (the computational simulation process is provided in the supporting materials), as listed in Table 3. The molar polarizability per unit volume (α/V) of the FPAIs was 0.863 and 0.925, which was higher than that of the FPEIs and FPEsIs. The amide group (-NHCO-) is a highly polar moiety with a large dipole moment (4.323 D for 6FDA-DABA and 4.610 D for 6FDA-PABA). Therefore, the FPAI films exhibit higher *D*_k_ and *D*_f_ values than both the FPEI and FPEsI films. Moreover, since the *D*_k_ of water is as high as approximately 80 under low-frequency electric fields and decreases slightly at higher frequencies, and the *D*_f_ of water increases from about 0.01 at low frequencies to approximately 0.484 at high frequencies [14,36,37,38], the water absorption of polymers has a significant impact on their *D*_k_ and *D*_f_. Accordingly, the high-dielectric properties observed in the FPAI films are consistent with the previously reported water absorption characterization results. The FPEsIs exhibited an ultra-low *D*_k_ of approximately 3.0 and low *D*_f_ values of ≈0.003 and ≈0.002, respectively. A comparison between APAB-6FDA and ABHQ-6FDA revealed that as the ester content increased, the *D*_k_ values remained nearly unchanged, whereas the *D*_f_ values showed a decreasing trend. This can be attributed to the ester groups, which are highly oriented and crystalline structural units. Incorporating them into the FPI backbone enhances the polymer’s molecular orientation and crystallinity, thereby restricting dipole moments and molecular chain mobility, which effectively reduces *D*_f_ [4,30,34,39,40]. Meanwhile, the increased ester content significantly enhanced the conformational rigidity of the polymer chains, as indicated by Kuhn segment parameter (*A***_fr_**) and radius of gyration (*R*_g_) values. The rigid polymer backbone restricted local molecular motions, such as vibrations and rotations, thereby reducing the ability of dipoles to reorient under high-frequency electric fields. This constrained dipole motion led to lower polarization relaxation energy dissipation, ultimately manifesting as a reduction in *D*_f_. Additionally, the increase in ester content markedly enhanced intermolecular interactions, as reflected by an increase in the cohesive energy density (CED). Stronger intermolecular forces not only stabilized the linear conformation of polymer chains but also restricted segmental motion, thereby reducing dipole mobility. This “locking effect” diminished the contribution of relaxation polarization at high frequencies, further lowering the *D*_f_ [33]. For the FPEIs (ODA-6FDA and TPE-6FDA), the *D*_k_ values were approximately 2.9 and 3.3, while the *D*_f_ values were 0.006 and 0.002, respectively. Similar to ester groups, increasing the ether content led to a rise in *D*_k_, whereas *D*_f_ decreased significantly. However, ether linkages serve as flexible bridging units, facilitating molecular orientation under high-frequency electric fields [31,32], thereby effectively reducing *D*_f_.

As the frequency increased, all the FPI films exhibited a slight decline in both *D*_k_ and *D*_f_, which aligns with theoretical models of polarization saturation and constrained molecular motion [41,42]. At frequencies above 10 GHz, the rapid oscillation of the electric field allows only the fastest polarization mechanisms (such as electronic and atomic polarization) to keep pace with field variations. In contrast, orientation polarization (dipole rotation) is significantly suppressed due to inertia, reducing its contribution. At high frequencies, the polarizability (α) of electronic and atomic polarization tends to stabilize, while the free volume of the material may increase due to restricted polymer chain motion, leading to a reduction in *D*_k_. At very high frequencies (e.g., 60 GHz), dipoles fail to complete full reorientation due to inertia, resulting in lower energy dissipation. At this stage, *D*_f_ is predominantly governed by the slight lag in electronic and atomic polarization, both of which exhibit minimal energy loss. Overall, FPAIs demonstrate potential for high-dielectric applications, while FPEsIs and FPEIs are more suitable for 5G/6G communication and other low-dielectric applications.

**Figure 4:**
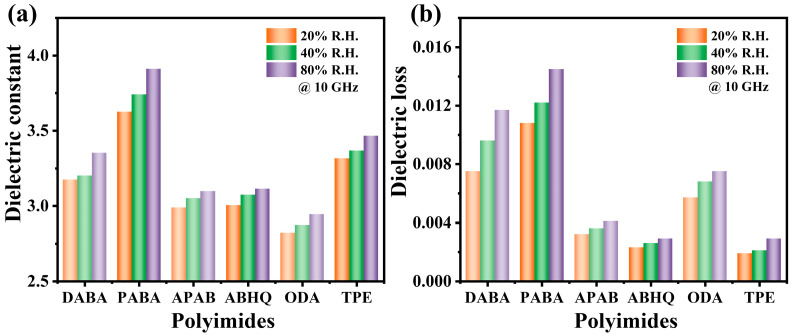
(**a**) RH dependence of *D*_k_ and (**b**) *D*_f_ for the six FPIs at 10 GHz.

Given the varying sensitivity of FPIs with different molecular structures to environmental humidity, it is essential to investigate their dielectric behavior under different humidity conditions. In this study, the effects of humidity (20%, 40%, and 80% RH) on the *D*_k_ and *D*_f_ of six FPI films with different functional groups were measured at 25 °C under a 10 GHz high-frequency electric field. The results are presented in Figure 4. A comparison of FPAIs, FPEsIs, and FPEIs revealed that the FPAIs exhibited the highest *D*_k_ and *D*_f_ values, as well as the greatest sensitivity to humidity. This behavior is attributed to the hydrophilic nature of amide groups, which readily form hydrogen bonds with water molecules in the environment, making the material highly susceptible to moisture absorption. Furthermore, the greater the number of amide groups, the higher the humidity sensitivity. Notably, the FPEsIs displayed exceptional stability in *D*_k_ and *D*_f_ across different humidity conditions. This stability is primarily attributed to the liquid crystalline nature of ester groups, which promotes molecular chain orientation and enhances local crystallinity, effectively hindering the penetration of water molecules into the PI matrix. In contrast, the PEIs exhibited slightly higher sensitivity to humidity than the FPEsIs, which is likely due to the weak hydrophilicity of ether groups. Overall, the humidity sensitivity of the investigated FPIs follows the trend FPAIs > FPEIs > FPEsIs, which is consistent with the trend observed in their water absorption properties.

**Figure 5:**
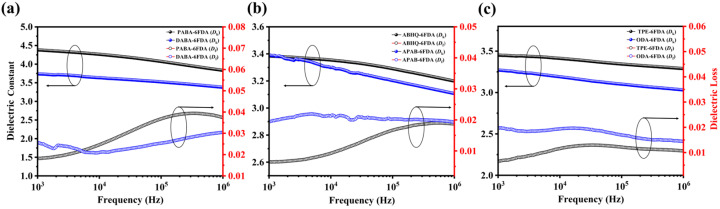
(**a**–**c**) Dielectric spectra of the FPI films.

Investigating the dielectric properties of PI films at low frequencies is equally important. To further explore this aspect, the dielectric performance of the synthesized FPI films was measured over a low-frequency range (10^3^–10^6^ Hz), and their dielectric spectra at different frequencies are presented in Figure 5. For all FPI films, the *D*_k_ decreases with increasing frequency. In the low-frequency region, dipolar orientation polarization plays a dominant role in the dielectric response. The polar functional groups on the polymer backbone have sufficient time to realign with the alternating electric field, resulting in high polarization ability and a relatively high dielectric constant. Among these, FPAIs exhibit the highest *D*_k_ values due to their strong polarity. As the frequency increases, the dipoles struggle to follow the rapid changes in the electric field, leading to a decrease in effective polarizability and consequently a reduction in *D*_k_. The *D*_f_ values, on the other hand, exhibit fluctuations with increasing frequency. However, the overall trend shows an initial slight increase followed by stabilization. This behavior is primarily attributed to the large dipole moments of highly polar functional groups (such as amide and ester groups), which require a longer response time to align with the electric field, leading to increased energy dissipation and a slight rise in *D*_f_ at lower frequencies [43]. In summary, regardless of the applied frequency, the incorporation of ester groups into the FPI backbone effectively reduces both *D*_k_ and *D*_f_ values, while the introduction of ether groups decreases *D*_f_ but slightly increases *D*_k_.

### 3.5. Optical Properties

**Figure 6:**
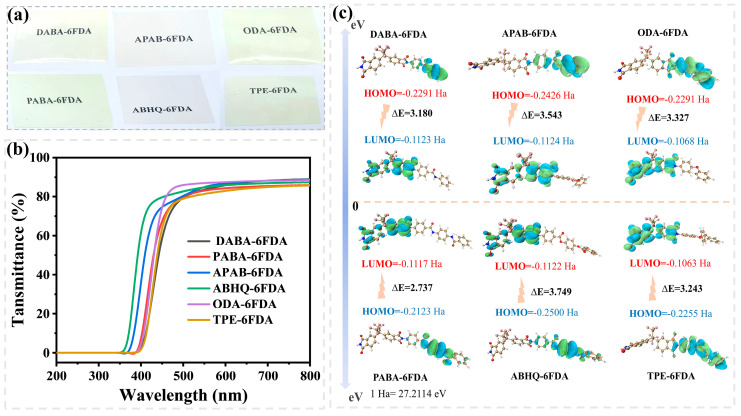
(**a**) Photographs of FPI films; (**b**) UV–Vis spectra of FPI films; (**c**) HOMO–LUMO gap energies of FPIs.

FPI films, particularly those containing CF_3_ groups, generally exhibit excellent colorless transparency, making them widely applicable in optoelectronic devices. The optical properties of the six FPI films were evaluated using UV–Vis spectrophotometry and a Yellowness Index (YI) meter, with the results summarized in Figure 6 and Table 4. The FPEsIs demonstrated the most outstanding optical performance, with a cut-off wavelength (*λ*_cut-off_) of 339 nm, and *T*_450 nm_ and *T*_550 nm_ values reaching 80% and 86%, respectively, while the YI was as low as 4.74. A comparison between APAB-6FDA and ABHQ-6FDA revealed that an increased ester group content in the FPI backbone enhances the colorless transparency of the films. This is attributed to the poor planarity of the ester group (Figure S1), which induces distortions in the polymer backbone, disrupts conjugation, and reduces electron cloud migration between the diamine and dianhydride moieties, thereby minimizing light absorption. Additionally, the LUMO–HOMO gap of the PEsIs was found to be larger than that of the other FPI structures (Figure 6c), with APAB-6FDA at 3.543 eV and ABHQ-6FDA at 3.749 eV. As the ester content increased, the widening of the bandgap led to a blue shift in the absorption edge, further reducing absorption in the visible region. For the FPAIs and FPEIs, relatively good colorless transparency was observed, with *λ*_cut-off_ values ranging from 372 to 387 nm, *T*_450 nm_ and *T*_550 nm_ in the ranges of 59–68% and 82–86%, respectively, and YI values between 8.71 and 9.56. However, as the content of amide and ether groups increased, the optical properties of the corresponding FPI films deteriorated. This is mainly due to hydrogen bonding interactions between the N–H in amide groups and adjacent C=O groups, which enhance interchain interactions and promote the formation of charge transfer complexes (CTCs). Furthermore, the electron-donating nature of the amide group facilitates charge transfer between diamine and dianhydride units, leading to a reduced LUMO–HOMO gap (DABA-6FDA: 3.180 eV; PABA-6FDA: 2.737 eV) and consequently a decline in optical performance. It is also noteworthy that the flexibility of ether linkages causes looser molecular packing and increased free volume within the polymer. However, the -C(CF_3_)_2_- group in 6FDA is a strong electron-withdrawing moiety, which can suppress CTC effects. When more ether linkages are introduced, their electron-donating effect may counteract the electron-withdrawing nature of F atoms, ultimately leading to a reduction in the bandgap (ODA-6FDA: 3.327 eV; TPE-6FDA: 3.243 eV).

### 3.6. Thermal and Mechanical Properties

**Figure 7:**
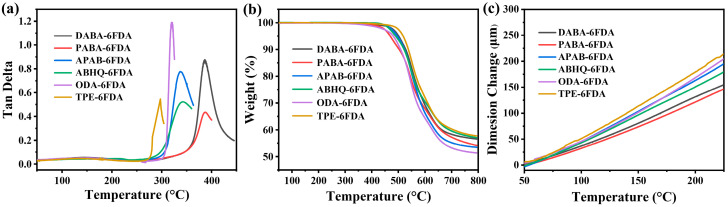
(**a**) DMA curve of FPI films; (**b**) TGA curve of FPI films; (**c**) TMA curve of FPI films.

The *T*_g_, thermal decomposition temperature, and thermal dimensional stability of the six FPI films were evaluated using DMA, TGA, and TMA, respectively. The corresponding data are summarized in Table 5. Figure 7a presents the Tan Delta curves obtained from DMA, where the peak values correspond to the *T*_g_ of the films. The *T*_g_ values of all the FPI films ranged from 296 °C to 388 °C. Among these materials, the FPAI films exhibited the highest *T*_g_ values compared to the FPEsIs and FPEIs. Specifically, PABA-6FDA and DABA-6FDA exhibited *T*_g_ values of 383 °C and 388 °C, respectively. This enhancement is primarily attributed to the introduction of hydrogen bonding in the amide groups, which strengthens intermolecular interactions and thus increases the *T*_g_ of the FPI films. This is supported by the results of polymer simulation calculations (Table 3), which show that the FPAIs possess higher values of *R*_g_ (39.85 and 49.04 Å), *A*_fr_ (2.71 and 2.84 Å), and CED (413.8 and 460.4 J/cm^3^) compared to the FPEsIs and FPEIs, indicating greater chain rigidity and stronger intermolecular interactions. In contrast, the FPEIs exhibited lower *T*_g_ values, with ODA-6FDA and TPE-6FDA showing *T*_g_ values of 320 °C and 296 °C, respectively. This reduction is due to the high flexibility of ether linkages, which reduces the rigidity of the polymer backbone, leading to a lower *T*_g_ for the FPEIs. For the FPEsIs, the *T*_g_ values of APAB-6FDA and ABHQ-6FDA were recorded as 337 °C and 344 °C, respectively. Additionally, due to the inherent rigidity of ester groups, the gradual elevation in ester group content induces a moderate enhancement in the *T*_g_ of FPI films.

The thermal stability of the films was evaluated using TGA, and the corresponding TGA curves of the six FPI films are presented in Figure 7b, with detailed data summarized in Table 5. Compared to conventional aromatic FPIs, these FPI films exhibit relatively lower thermal stability due to the presence of a higher proportion of single-bonded functional groups, such as -C(CF_3_)_2_-, ester, amide, and ether groups. The measured *T*_d_^5%^ and *T*_d_^10%^ values ranged from 467 °C to 516 °C and 495 °C to 539 °C, respectively. However, given the inherently high thermal stability of FPI materials, these FPI films still possess sufficient thermal resistance to meet the requirements of 5G communication and optoelectronic applications. Additionally, at 800 °C, the residual mass fraction of these FPI films ranged from 50.2% to 54.2%, demonstrating their high char yield and excellent thermal stability. The dimensional stability of the six FPI films was further analyzed via TMA, and the TMA curves are displayed in Figure 7c, with the CTE values listed in Table 5. The FPAIs exhibited the lowest CTE, with DABA-6FDA and PABA-6FDA showing a CTE of 14.7 and 11.2 ppm/K, respectively. This reduction in CTE is attributed to hydrogen bonding interactions between amide groups, which enhance intermolecular interactions and thus improve dimensional stability. In contrast, the FPEIs (ODA-6FDA and TPE-6FDA) exhibited the highest CTE, at 49.5 and 52.5 ppm/K, respectively. This is primarily due to the flexibility of ether linkages and their bent conformation, which reduces the rigidity of the polymer backbone. The CTE values of the FPEsIs (APAB-6FDA and ABHQ-6FDA) were 37.5 and 32.3 ppm/K, respectively, which can be attributed to the molecular architecture of ester groups exhibiting inherent rigidity and aggregation orientation. These structural characteristics endow FPEsIs with superior thermal dimensional stability compared to FPEIs. Furthermore, the incremental incorporation of ester functionalities was observed to induce a further reduction in the CTE of FPIs.

To further investigate their mechanical performance, six FPI films containing different functional groups were evaluated using a universal testing machine, with the results summarized in Table 5. Both the FPEsIs and FPEIs demonstrated good mechanical properties, with ultimate tensile strengths of 149.8–167.2 MPa and 152.5–161.6 MPa, Young’s moduli of 2.5–2.6 GPa and 2.1–2.3 GPa, and elongations at break of 9.5–11.3% and 12.5–14.3%, respectively. In contrast, the FPAIs exhibited significantly superior mechanical properties, owing to hydrogen bonding interactions that enhance intermolecular forces within the PI matrix. These films achieved remarkably higher ultimate tensile strengths of 219.4–248.1 MPa, Young’s moduli of 3.1–3.4 GPa, and elongations at break of 16.7–18.6%. Overall, the incorporation of amide groups into the PI backbone is more effective in enhancing mechanical performance than that of ether or ester groups.

## 4. Conclusions

This study first synthesized a diamine monomer (PABA) and then selected five commercial diamines for homopolymerization with 6FDA dianhydride via thermal imidization, producing six FPI films (FPAIs, FPEsIs, and FPEIs) containing distinct functional groups. The systematic characterization of the dielectric, optical, thermal, and mechanical properties revealed that the FPAIs exhibited the highest *D*_k_ and *D*_f_ across both high- and low-frequency ranges. In contrast, the FPEsIs and FPEIs demonstrated ultra-low-dielectric performance, particularly achieving a *D*_f_ as low as ~0.002 at high frequencies, with *D*_k_ values ranging from 2.8 to 3.3, alongside excellent dielectric humidity stability, underscoring their significant application potential in 5G/6G communication systems. Regarding optical performance, the FPEsI films displayed optimal colorless transparency, with a *T*_450 nm_ of 80% and a YI of 4.74, whereas the FPAIs and FPEIs exhibited slightly inferior optical properties. In terms of thermal and mechanical performance, the FPAIs demonstrated superior thermomechanical stability due to hydrogen bonding interactions from amide linkages, featuring a higher *T*_g_ (388 °C), lower CTE (11.2 ppm/K), and enhanced *σ* (248.1 MPa), *E* (3.4 GPa), and *ε* (18.6%). The comparative analysis between the FPEsIs and FPEIs indicated that the FPEsIs possessed a lower CTE, higher *E*, and reduced *ε*. Finally, molecular dynamics simulations and quantum chemical calculation were employed to comprehensively investigate the mechanistic influence of functional group structures on the dielectric and optical properties of the FPI films, providing essential theoretical guidance for the rational design and application of FPIs in high-frequency communication and optoelectronic device fields.

## Figures and Tables

**Table 1:** Physical parameters of the FPIs.

Polymer	*M*_n_ (×10^4^) *^a^*	*M*_w_ (×10^4^) *^a^*	PDI	*A* (%)	CA (°)
DABA-6FDA	8.0	15.2	1.9	2.27	89.5 ± 1.8
PABA-6FDA	5.3	10.5	2.0	2.65	88.4 ± 0.9
APAB-6FDA	5.9	11.2	1.9	0.98	93.1 ± 1.7
ABHQ-6FDA	4.8	9.2	1.9	0.79	96.1 ± 1.3
ODA-6FDA	4.7	9.8	2.1	1.62	89.7 ± 1.5
TPE-6FDA	5.6	13.4	2.4	1.94	89.9 ± 1.3

*^a^* Molecular weight of the polymers; unit: g mol^−1^.

**Table 2:** Dielectric properties of FPI films at high frequency.

Polymers	*D*_k_ (Dielectric Constant)			*D*_f_ (Dielectric Loss)		
	10 GHz	40 GHz	60 GHz	10 GHz	40 GHz	60 GHz
DABA-6FDA	3.201	3.192	3.190	0.0096	0.0095	0.0092
PABA-6FDA	3.741	3.721	3.699	0.0122	0.0120	0.0120
APAB-6FDA	3.050	3.032	3.026	0.0036	0.0034	0.0031
ABHQ-6FDA	3.073	3.053	3.014	0.0026	0.0025	0.0023
ODA-6FDA	2.872	2.871	2.868	0.0068	0.0065	0.0063
TPE-6FDA	3.367	3.342	3.339	0.0021	0.0022	0.0020

**Table 3:** Physical parameters obtained from MD simulations of FPIs.

FPI	*R*_g_ (Å) *^a^*	*A*_fr_ (Å) *^b^*	Dipole Moment (D)	α/V (a.u.× mol/cm^3^) *^c^*	CED (J/cm^3^) *^d^*	FFV (%) *^e^*	Density (g/cm^3^)
DABA-6FDA	39.85	2.71	4.323	0.863	413.8	24.79	1.341
PABA-6FDA	49.04	2.84	4.610	0.925	460.4	23.30	1.330
APAB-6FDA	33.89	1.47	2.410	0.859	396.9	23.13	1.372
ABHQ-6FDA	39.10	1.86	3.404	0.884	406.5	22.51	1.353
ODA-6FDA	29.07	1.19	2.701	0.855	370.9	22.99	1.370
TPE-6FDA	30.14	1.37	2.773	0.867	358.6	23.62	1.328

*^a^* Radius of gyration. *^b^* Kuhn segment parameter. *^c^* Volume polarizability. *^d^* Cohesive energy density. *^e^* Fractional free volume, FFV = (V − 1.3*V*_W_)/*V*.

**Table 4:** The optical parameters of the FPI films.

Polymers	*d* (μm) *^a^*	*λ*_cut-off_ (nm) *^b^*	*T*_450_ (%) *^c^*	*T*_550_ (%) *^d^*	YI *^e^*	∆*E ^f^*
DABA-6FDA	33	372	59	84	8.71	3.180
PABA-6FDA	34	387	68	83	9.46	2.737
APAB-6FDA	46	357	75	85	7.45	3.543
ABHQ-6FDA	40	339	80	86	4.74	3.749
ODA-6FDA	38	387	68	86	9.27	3.327
TPE-6FDA	29	372	64	82	9.56	3.243

*^a^* Film thickness. *^b^* Cut-off wavelength. *^c^* Optical transmittance at 450 nm. *^d^* Optical transmittance at 550 nm. *^e^* YI means Yellowness Index. *^f^* LUMO–HOMO gap.

**Table 5:** Thermal and mechanical performance of FPI films.

Polymers	*T*_g_ (°C) *^a^*	*T*_d_^5%^ (°C) *^b^*	*T*_d_^10%^ (°C) *^c^*	*R*_w_ (%) *^d^*	CTE (ppm/K) *^e^*	*σ* (MPa) *^f^*	*E* (GPa) *^g^*	*ε* (%) *^h^*
DABA-6FDA	382	471	514	52.7	14.7	219.4 ± 9.8	3.1 ± 0.3	16.7 ± 3.7
PABA-6FDA	388	469	503	54.2	11.2	248.1 ± 11.2	3.4 ± 0.3	18.6 ± 4.5
APAB-6FDA	337	475	514	51.8	37.5	149.8 ± 9.5	2.6 ± 0.4	11.3 ± 1.8
ABHQ-6FDA	344	467	495	50.4	32.3	167.2 ± 7.4	2.5 ± 0.3	9.5 ± 1.6
ODA-6FDA	320	516	539	51.5	49.5	152.5 ± 6.8	2.1 ± 0.1	12.5 ± 1.8
TPE-6FDA	296	489	521	50.2	52.5	161.6 ± 8.6	2.3 ± 0.4	14.3 ± 3.2

*^a^ T*_g_: glass transition temperature tested by DMA. *^b^ T*_d_^5%^: temperatures at 5% weight loss. *^c^ T*_d_^10%^: temperatures at 10% weight loss. *^d^* Residual weight retention when heated to 800 °C in N_2_. *^e^* CTE: measured with TMA in the temperature range of 50–200 °C. *^f^* Tensile strength. *^g^* Young’s moduli. *^h^* Elongation at break.

## Data Availability

The original contributions presented in the study are included in the article/Supplementary Materials, further inquiries can be directed to the corresponding author.

## Supplementary Materials

The following supporting information can be downloaded at: https://www.mdpi.com/article/10.3390/polym17111505/s1, References [44,45,46,47,48,49,50] are cited in the supplementary materials. Table S1: Solubility of FPI powders; Figure S1: The dihedral angle of FPIs. The Supporting Information is available free of charge.
