# Supplementary material for: Effect of Diamine Monomers with Varied Backbone Structures on Dielectric and Other Comprehensive Properties of Fluorinated Polyimide Films
Source: Polymers (Basel). 2025 May 28;17(11):1505. doi: 10.3390/polym17111505 (PMC12157939; doi:10.3390/polym17111505)
Supplement: Supplementary file 1 [file polymers-17-01505-s001.zip › polymers-3669790-supplementary.pdf]

Supplementary Materials

Effect of Diamine Monomers with Varied Backbone Structures on Dielectric and Other Comprehensive Properties of Fluorinated Polyimide Films

Wenhao Xu [1], Xiaojie He [2], Yu Zhou [2], Lan Jiang [1], Weiyou Yang [1], Qinghua Lu [3*],

Peng Xiao [1,2,*]

1. Institute of Micro/Nano Materials and Devices, Ningbo University of Technology, Ningbo 315211, P.R. China
2. School of Chemical Science and Engineering, Tongji University, Siping Road No. 1239, Shanghai, 200092 China
3. State Key Laboratory of Micro-Nano Engineering Science, Shanghai Jiao Tong University, Dongchuan Road No. 800, Shanghai, 200240 China

E-mail: pengxiao2020@tongji.edu.cn (Peng Xiao).

Contents

1. **Simulation details**

   1.1 Molecular dynamics simulations

   1.2 Quantum chemical calculation

2. **Table S1 Solubility of FPI powders**

3. **Figure S1. The dihedral angle of FPIs**

4. **References**

1. Simulation details

1.1 Molecular dynamics simulations

The molecular dynamic simulation was performed in Materials Studio 2019. Five amorphous cells with 12 structurally optimized PI chains were established by the Amorphous Cell module. Geometry optimization of these models was carried out using the smart algorithm with maximum interactions of 50000 steps in the Forcite Module. 50 ps NPT dynamics was then performed to obtain a structure of reasonable density. Then, 400 ps molecular dynamics was performed under the NVT ensemble at 298 K to eliminate internal stress, Finally, 800 ps molecular dynamics under the NPT ensemble at 0.0001 GPa and 298 K was carried out at the initial speed of the previous step to obtain a stable equilibrium density. COMPASSII force field was utilized in the MD simulations. On this basis, some relevant parameters were calculated.

The radius of gyration (R_g) represents the root mean square distance of each molecule from its center of mass. The radius of gyration was calculated as:

$$\boldsymbol{R_g^2} = \frac{\sum_{i=1}^{N} m_i r_i^2}{\sum_{i=1}^{N} m_i} \tag{1}$$

Where r_i (Å) is the distance from each atom to the center of mass, m_i (g) is the mass of atom i and N is the number of atoms[1].

The cohesive energy density (CED) refers to the energy required for gasification per mole of material per unit volume to overcome intermolecular forces, which includes the van der Waals CED and electrostatic CED[2].

The adequate measure of conformational rigidity is the value of the Kuhn segment parameter:

$$A_{\mathrm{fr}} = \lim_{n \to \infty} \left(\frac{\langle h^2 \rangle}{n l_0} \right) \tag{2}$$

where h^2 is the mean square distance between the chain ends, n is the number of the repeat units and l_0 is the contour length of the repeat unit[3].

The fractional free volume (FFV) is defined as the specific volume of the membrane matrix divided by the free volume. The Atom Volumes & Surfaces module of MS was employed to generate various surfaces. The FFV of the simulation boxes

was calculated as:

$$\mathrm{FFV} = \frac{V - V_0}{V} \tag{3}$$

$$V_0 = 1.3 \times V_W \tag{4}$$

Where V represents the volume of simulation box, the volume of a van der Waals system is denoted by V_W and an indication of V_0 signifies the volume that the polymer chain occupies[4].

1.2 Quantum chemical calculation

The HOMO-LUMO energy levels[5] for six FPIs were performed using Gaussian 16[6] following a rigorous computational protocol. First, geometry optimization and vibrational frequency analysis were conducted at the B3LYP/6-311+G(d) level to ensure stable ground-state configurations. This step generated optimized geometries and verified the absence of imaginary frequencies in the output files to confirm true energy minima. Subsequently, under the same functional and basis set, the single-point energy of each structure is calculated. After the calculation is completed, the energy of the HOMO and LUMO orbitals can be viewed through GaussView6.0[7].

Table S1 Solubility of FPI powders[a]

Polyimides	DMAc	DMF	NMP	*m*-Cresol	DCM	THF	MeOH
DABA-6FDA	++	++	++	++	++	+	-
PABA-6FDA	++	++	++	++	++	+	-
APAB-6FDA	-	-	-	-	-	-	-
ABHQ-6FDA	-	-	-	-	-	-	-
ODA-6FDA	++	++	++	++	++	+	-
TPE-6FDA	++	++	++	++	++	+	-

[a] Key: at room temperature: ++, readily soluble; +, soluble; −, insoluble.

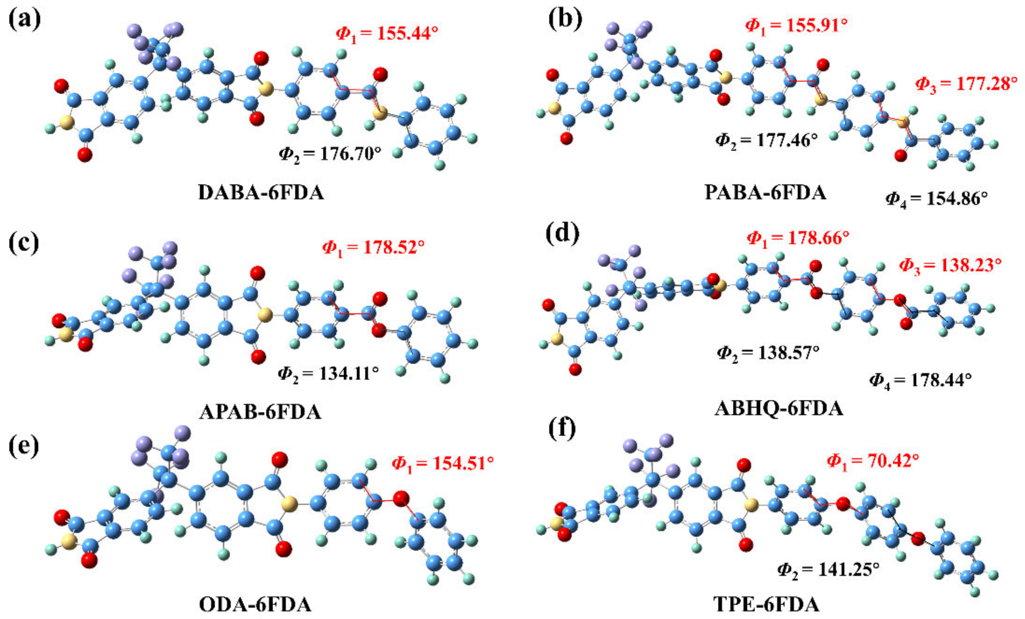

Figure. S1. The dihedral angle of FPIs

References

1. Eftekhari, A.; Amin, J. S.; Zendehboudi, S., A molecular dynamics approach to investigate effect of pressure on asphaltene self-aggregation. *Journal of Molecular Liquids* **2023**, 376, 121347.
2. Chen, F.; Liu, F.; Du, X., Molecular dynamics simulation of crosslinking process and mechanical properties of epoxy under the accelerator. *Journal of Applied Polymer Science* **2023**, 140 (2), e53302.
3. Hamciuc, C.; Ronova, I. A.; Hamciuc, E.; Bruma, M., The effect of the rotation hindrance on physical properties of some heterocyclic polyamides containing pendent imide groups. *Die Angewandte Makromolekulare Chemie* **1998**, 254 (1), 67-74.
4. Salahshoori, I.; Mohseni, A.; Namayandeh Jorabchi, M.; Ghasemi, S.; Afshar, M.; Wohlrab, S., Study of modified PVDF membranes with high-capacity adsorption features using Quantum mechanics, Monte Carlo, and Molecular Dynamics Simulations. *Journal of Molecular Liquids* **2023**, 375, 121286.
5. Wakita, J.; Sekino, H.; Sakai, K.; Urano, Y.; Ando, S., Molecular Design, Synthesis, and Properties of Highly Fluorescent Polyimides. *The Journal of Physical Chemistry B* **2009**, 113 (46), 15212-15224.
6. Gaussian 16, Revision B.01, M. J. Frisch, G. W. Trucks, H. B. Schlegel, G. E. Scuseria, M. A. Robb, J. R. Cheeseman, G. Scalmani, V. Barone, G. A. Petersson, H. Nakatsuji, X. Li, M. Caricato, A. V. Marenich, J. Bloino, B. G. Janesko, R. Gomperts, B. Mennucci, H. P. Hratchian, J. V. Ortiz, A. F. Izmaylov, J. L. Sonnenberg, D. Williams-Young, F. Ding, F. Lipparini, F. Egidi, J. Goings, B. Peng, A. Petrone, T. Henderson, D. Ranasinghe, V. G. Zakrzewski, J. Gao, N. Rega, G. Zheng, W. Liang, M. Hada, M. Ehara, K. Toyota, R. Fukuda, J. Hasegawa, M. Ishida, T. Nakajima, Y. Honda, O. Kitao, H. Nakai, T. Vreven, K. Throssell, J. A. Montgomery, Jr., J. E. Peralta, F. Ogliaro, M. J. Bearpark, J. J. Heyd, E. N. Brothers, K. N. Kudin, V. N. Staroverov, T. A. Keith, R. Kobayashi, J. Normand, K. Raghavachari, A. P. Rendell, J. C. Burant, S. S. Iyengar, J. Tomasi, M. Cossi, J. M. Millam, M. Klene, C. Adamo, R. Cammi, J. W. Ochterski, R. L.

Martin, K. Morokuma, O. Farkas, J. B. Foresman, and D. J. Fox, Gaussian, Inc., Wallingford CT, 2016.

7. GaussView, Version 6, Roy Dennington, Todd A. Keith, and John M. Millam, Semichem Inc., Shawnee Mission, KS, 2016.
